# Fish Oil Has Beneficial Effects on Allergen-Induced Airway Inflammation and Hyperreactivity in Mice

**DOI:** 10.1371/journal.pone.0075059

**Published:** 2013-09-06

**Authors:** Thereza Cristina Lonzetti Bargut, Tatiana Paula Teixeira Ferreira, Julio Beltrame Daleprane, Marco Aurélio Martins, Patrícia Machado Rodrigues Silva, Marcia Barbosa Aguila

**Affiliations:** 1 Laboratory of Morphometry, Metabolism and Cardiovascular Disease, Biomedical Center, Institute of Biology, State University of Rio de Janeiro, Rio de Janeiro, Rio de Janeiro, Brazil; 2 Laboratory of Inflammation, Oswaldo Cruz Institute, Oswaldo Cruz Foundation (FIOCRUZ), Rio de Janeiro, Rio de Janeiro, Brazil; Research Center Borstel, Germany

## Abstract

**Background:**

Fish oil (FO) is rich in n-3 polyunsaturated fatty acids (PUFA), which have been suggested to be anti-inflammatory and are associated with improvement of several inflammatory diseases. In this study, we investigated the influence of FO on allergen-induced lung inflammation and airway hyperreactivity in mice.

**Methods:**

Male A/J mice were fed either a standard-chow (SC) or a FO diet (FO) for 8 weeks. After 4 weeks, each group was further randomized for ovalbumin (SC-OVA and FO-OVA) or saline (SC-SAL and FO-SAL) challenge. Resistance and elastance were measured at baseline and after aerosolized methacholine, 24h after the last challenge. Bronchoalveolar lavage (BAL) was performed for leukocyte counts. Lung tissue mucus deposition, peribronchiolar matrix deposition and eosinophil infiltration were quantified. Serum immunoglobulin E (IgE) and IgG1 (ref 2.2), lung IL-4, IL-5, IL-10, IL-13, IL-17, INFγ and eotaxin-1 and 2 were detected by ELISA and nuclear factor kappa B (NFκB), GATA-3 and peroxisome proliferator-activated receptor gamma (PPARγ) expression was measured by Western blot.

**Results:**

Levels of serum IgE and IgG1 were significantly higher in OVA sensitized mice. OVA challenge resulted in increased eosinophil infiltration, increased inflammatory cytokine production, peribronchiolar matrix and mucus deposition and airway hyperreactivity to aerosolized methacholine. Elevated lung NFκB and GATA-3 expression was noted in OVA-challenged mice. These changes were attenuated in mice fed with FO diet. Higher PPARγ expression was also detected in the lungs from the FO-fed groups.

**Conclusion:**

Our results demonstrate that FO intake attenuated classical asthma features by suppressing the systemic sensitization, thus providing evidence that FO might be a prophylactic alternative for asthma prevention.

## Introduction

Asthma is an inflammatory, chronic airway disease that is characterized by structural and functional changes, and its prevalence is widespread throughout the world [1]. The bronchial asthmatic response is based on a type 2 T helper cytokine (Th2) immune profile with leukocyte infiltration, particularly consisting of eosinophils, which are associated with pulmonary remodeling, goblet cell hyperplasia and mucus hyperproduction [2]. This process is controlled by inflammatory mediators such as cytokines and chemokines, which cause airway hyperreactivity (AHR) and airflow obstruction [3]. However, there is no cure for asthma, and its control requires using anti-inflammatory agents, especially glucocorticoids, which have a broad spectrum of adverse effects. Additionally, 5-10% of asthmatic patients are resistant to glucocorticoids, which supports the need to search for new therapies [4].

Fish oil (FO) is rich in n-3 polyunsaturated fatty acids (PUFA), which include eicosapentaenoic acid (EPA) and docosahexaenoic acid (DHA) [5]. EPA and DHA ingestion partially substitute for cell membrane arachidonic acid (a n-6 PUFA) and compete for its degradation enzymes [6]. These mechanisms reduce 2 and 4-series eicosanoid production and enhance 3 and 5-series eicosanoid production, which display less pro-inflammatory activity [7]. Studies from our laboratory demonstrated that FO intake directly diminished cytokine production [8,9] through effects on transcription factors that control inflammatory responses, such as nuclear factor kappa B (NFκB) [10] and peroxisome proliferator-activated receptor (PPAR)-γ [11].

Clinical trials have demonstrated that FO intake reduces biomarkers and improves lung function in asthmatic children [12,13]. Similar effects were observed in adults, with diminished 2-series prostaglandin, 4-series leukotriene, interleukin-1β and tumor necrosis factor (TNF)-α concentrations, thus reducing the necessity for bronchodilators [14]. FO diminished oxidative stress markers in allergen-challenged mice, though its effects on other changes in lung tissue, inflammatory status and function remain uncertain [7,15].

The prophylactic effects of FO intake on allergen-induced airway inflammation in actively sensitized mice have not been studied. Thus, this is the aim of the present study.

## Material and Methods

### Animals and diet

Male A/J mice (18-20 g) were obtained from Oswaldo Cruz breeding and were maintained under controlled conditions (20 ± 2^°^C, 60 ± 10% humidity and 12 h dark/light cycle) with free access to food and water. Animals were fed a standard chow (SC; 7% wt/wt soybean oil) or a fish oil diet (FO; 6.3% wt/wt FO + 0.7% wt/wt soybean oil) for 8 weeks (Table 1). All diets were elaborated with purified nutrients by PragSoluções (Jau, São Paulo, Brazil) and were in accordance with the American Institute of Nutrition’s recommendation (AIN 93G) [16]. FO was purchased from Sigma-Aldrich (FO from menhaden fish - Sigma-Aldrich Co., St Louis, MO, USA) and contains 12.9% of EPA and 12% of DHA (EPA/DHA ratio = 1.075). Food intake was measured daily and body mass was assessed weekly. All of the procedures were examined and approved by the Animal Ethics Committee of the Oswaldo Cruz Foundation (CEUA-FIOCRUZ, L034/09) (Rio de Janeiro, Brazil).

**Table 1:** Composition and energy content of the standard chow (SC) (AIN 93G) and the fish oil (FO) (AIN 93G-based diet) diets.

	Diet	
Content (g/Kg)	SC	FO
Casein (≥ 85% of protein)	200.0	200.0
L-Cystine	3.0	3.0
Cornstarch	529.486	529.486
Sucrose	100.0	100.0
Soybean oil	70.0	7.0
Fish oil	-	63.0
Fiber	50.0	50.0
Vitamin mix*	10.0	10.0
Mineral mix*	35.0	35.0
Choline	2.5	2.5
Antioxidant	0.014	0.014
Total mass	1,000. 0	1,000. 0
Energy content (Kcal/kg)	3960	3960
Carbohydrates (% Energy)	64	64
Proteins (% Energy)	19	19
Lipids (% Energy)	17	17

* Mineral and vitamin mixtures are in accordance with AIN 93G.

### Animal preparation and experimental protocol

After four weeks of the diet, the 2 groups were randomized to be sensitized and challenged with ovalbumin (albumin from chicken egg white - A5503, Sigma-Aldrich, St. Louis, MO, USA) and saline. Mice were sensitized subcutaneously with a mixture of 50 µg ovalbumin (OVA) and 5 mg aluminum hydroxide on days 0 and 14. On days 21 and 22, animals were intranasally challenged with 25 µg OVA. Control animals received saline (SAL). Animals continued to be given the respective diets during sensitization and challenge periods. The analyses were performed 24 hours after the last challenge. The experiments were done twice and data shown are representative of one experiment. In the particular case of lung function, three experiments were done and data shown are representative of two of them.

### Lung function and airway hyperreactivity

Animals were anesthetized (Nembutal 60 mg/kg i.p.) and tracheotomized for pulmonary function and hyperreactivity assessment in a FinePoint R/C Buxco Platform (Buxco Electronics, Sharom, CT, USA) 24 hours after the last challenge (day 23). Airflow and transpulmonary pressure were recorded using a Buxco Pulmonary Mechanics Processing System (Buxco Electronics, Wilmington, NC, USA), which was also used to calculate airway resistance (cmH _2_O/ml/s) and dynamic compliance (ml/cmH _2_O). Analog signals from the computer were digitized using a Buxco analog to digital converter (Buxco Electronics). Mice were stabilized for 5 minutes, and increasing methacholine concentrations (3, 9, and 27 mg/ml) were aerosolized for 5 minutes each. Baseline resistance and dynamic compliance were assessed with aerosolized phosphate-buffered saline (PBS). The results were expressed as the mean absolute values of lung resistance and elastance (calculated as the inverse of compliance values - cmH _2_O/ml) responses recorded during the 5 minutes after methacholine aerosol administration.

### Bronchoalveolar lavage (BAL)

Lungs were washed via tracheal tube with PBS solution (1 ml) containing EDTA (10 mM). Samples were centrifuged at 300 x g for 10 minutes. The supernatant was used for chemokine analysis, and the pellet was resuspended in 0.25 ml PBS. Total leukocyte numbers were measured in Neubauer chambers using light microcopy after diluting the samples in Türk solution (2% acetic acid). Differential cell counts were performed with cytospin smears using the May-Grünwald Giemsa method [17].

### Lung histology and morphometry

A laparotomy was performed immediately after the BAL. The abdominal aorta and vena cava were sectioned. The left lung was removed, fixed by immersion in 3% buffered formaldehyde. After fixation, the lung was cut longitudinally, imbedded in Paraplast (Sigma-Aldrich Co., St Louis, Mo., USA) and 3-µm thick sections were obtained for further analyses by means of light microscopy. Periodic acid-Schiff and Gomori Trichrome staining was performed to quantify mucus and extracellular matrix deposition. Measurements were made with video-microscopic system (LC Evolution camera, Olympus BX51 microscope) provided with an integrating eyepiece with a known area (10^4^ µm^2^) at a maginification of 400X). Nine to ten distal airways per lung were analyzed using Image-Pro Plus version 7.01 software (Media Cybernetics, Silver Spring, MD, USA) and only airways (bronchiole) with dimensions fitting in the counting frame area were considered [18]. We evaluated airway inflammatory cell infiltrates with Sirius Red pH 10.2 and employed a test system made up of 100 grid lines for the analysis. A total of 10-20 fields were analyzed per lung at a final magnification of 1,000x. Eosinophils present in the airway wall were counted in three randomly selected areas and were expressed as cells/unit area (10^4^ µm^2^).

### Serum anti-OVA IgE and IgG1 measurement

Blood was taken by cardiac puncture under light ether anesthesia 19 days after sensitization. After blood coagulation, individual sera were collected and stored at -20^o^C until use. Anti-OVA IgE and IgG1 were measured by means of enzyme-linked immunosorbent assays (ELISA) (Cayman Chemical Company, Ann Arbor, Michigan, USA and BioVendor Research and Diagnostic Products, Asheville, USA, respectively) according to the instructions of the manufacturer.

### Cytokine and chemokines measurement

Murine IL-4, IL-5, IL-10, IL-13, IL-17, INFγ and eotaxin-1 and -2 levels were measured in right lung tissue samples by means of ELISA technique using commercial Duo Set kits R&D Systems (Minneapolis, USA) following the instructions of the manufacturer.

### Western blot

Lung tissue was homogenized as described previously [19]. Briefly, protein was quantified, and 50 µg total protein was loaded on 10% SDS-polyacrylamide gels and blotted onto nitrocellulose membranes. Nonspecific binding was blocked with 5% (w/v) skim milk powder in T-TBS for 1 hour followed by incubation with GATA-3 1:500 (50 kDa; SC-22206; Santa Cruz Biotechnology), NFκB p65 1:500 (nuclear factor kappa B; 65 kDa; SC-372; Santa Cruz Biotechnology), PPARγ 1:1000 (peroxisome proliferator activated-receptor gamma; 54 kDa; SC-7273; Santa Cruz Biotechnology) or β-actin 1:2000 (43 kDa; SC-47778; Santa Cruz Biotechnology) antibodies overnight at 4°C. Blots were then incubated with appropriate horseradish peroxidase-conjugated secondary antibodies followed by enhanced chemiluminescence detection. Band intensities were quantified by densitometry using ImageJ 1.3 software (NIH, USA).

### Data analysis

The values are shown as the means and standard error of the mean (SEM). In the cases where we could confirm homocedasticity of variances, comparisons among groups were made using analysis of variance (ANOVA) followed by the Holm-Sidak post-hoc test. In each case, a *P*-value ≤ 0.05 was considered to be statistically significant. All of the analyses were performed using Graph Pad Prism version 6.01 for Windows (La Jolla, CA, USA).

## Results

There were no differences in food intake (SC-SAL: 2.67±0.03 g SC-OVA: 2.70±0.003 g; FO-SAL: 2.78±0.004 g; FO-OVA: 2.75±0.004 g) or body mass (SC-SAL: 25.05±0.36 g SC-OVA: 24.44±0.79 g; FO-SAL: 25.13±0.84 g; FO-OVA: 24.97±0.63 g) among the experimental groups during the experiment.

### Effect of FO administration on lung leukocyte recruitment

**Figure 1:**
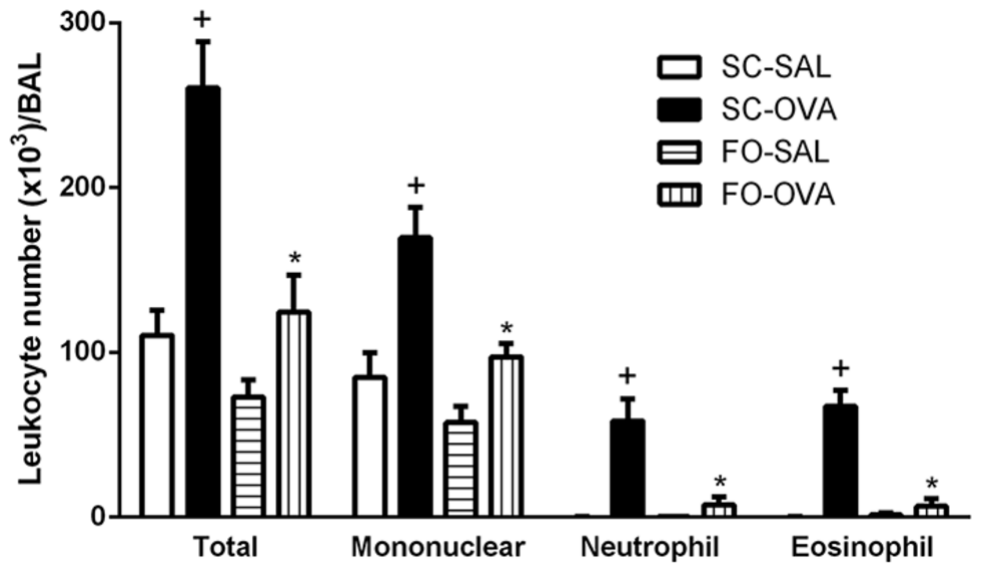
Effect of FO intake on allergen-evoked leukocyte infiltration in BAL fluid from A/J mice. Sensitized, saline-challenged (SC-SAL); sensitized, ovalbumin-challenged (SC-OVA); sensitized, saline-challenged with fish oil (FO-SAL) and sensitized, ovalbumin-challenged with fish-oil (FO-OVA). The analyses were performed 24 hours after the last challenge. In the signaled cases, *P*<0.05 compared with the SC-SAL group (+) and the SC-OVA group (*) (one-way ANOVA and post-hoc Holm-Sidak test). Values are the means ± S.E.M. and are representative of 1 experiment (n=5 per group).

Antigen challenge of actively sensitized mice (SC-OVA) increased the total BAL leukocyte counts 24 hours after the last challenge compared with control (SC-SAL) mice (+136%, *P*<0.0001). This elevation was because of increased mononuclear cells (2-fold increase, *P*=0.0018), neutrophils (more than 400-fold increase, *P*<0.0001) and eosinophils (more than 500-fold increase, *P*<0.0001). FO intake by sensitized, challenged mice (FO-OVA) inhibited total BAL leukocyte infiltration (-52%, *P*=0.0002), including mononuclear cells, neutrophils and eosinophils (*P*=0.0029, *P*<0.0001 and *P*=0.0002, respectively) (Figure 1).

**Figure 2:**
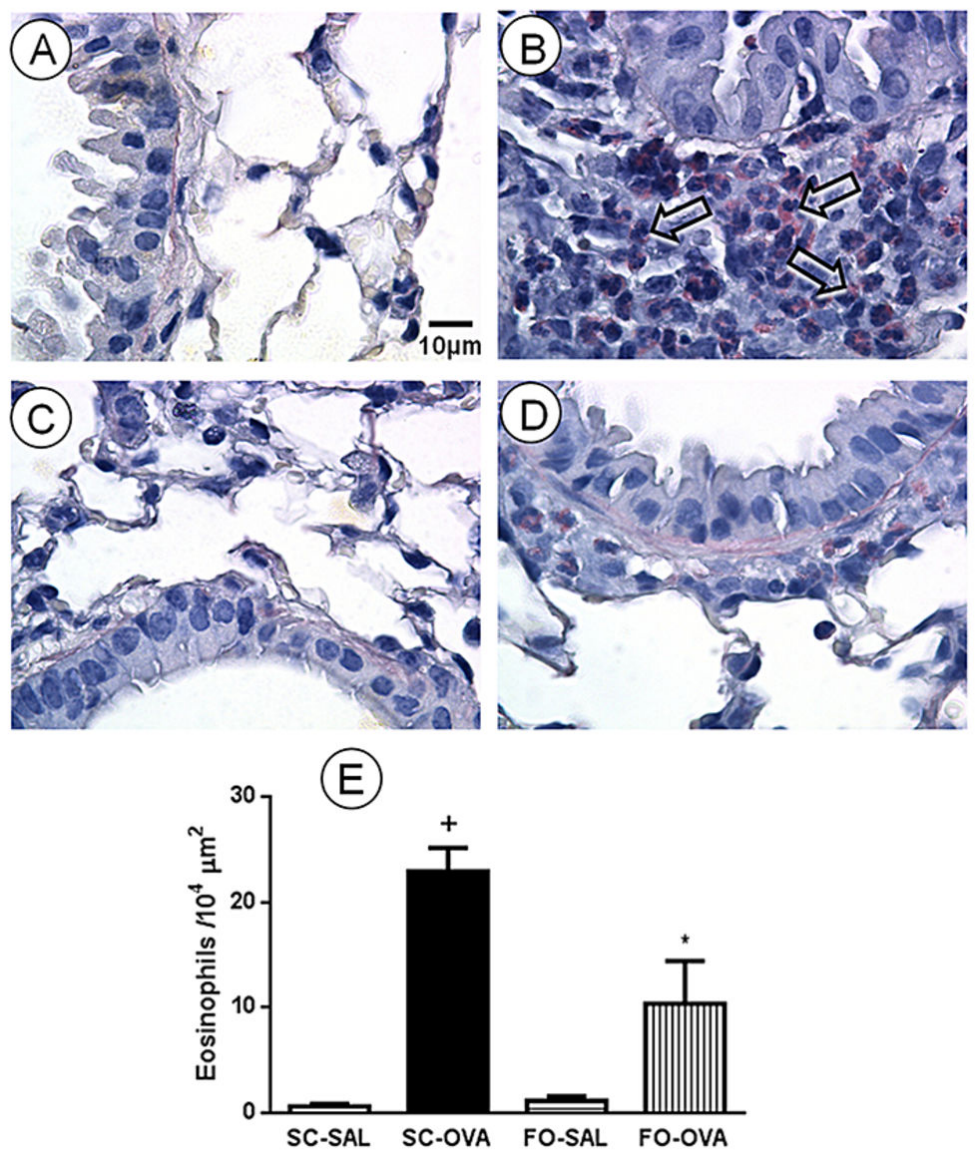
Effect of FO intake on allergen-evoked eosinophil lung tissue infiltration of A/J mice. Photomicrographs were taken of representative airways from (A) sensitized, saline-challenged (SC-SAL); (B) sensitized, ovalbumin-challenged (SC-OVA); (C) sensitized, saline-challenged with fish oil (FO-SAL) and (D) sensitized, ovalbumin-challenged with fish oil (FO-OVA). (E) Peribronchiolar eosinophil number was determined in lung sections by morphometric analyses. The analyses were performed 24 hours after the last challenge. Slides were stained with Sirius Red. Arrows indicate representative eosinophils. In the signaled cases, *P*<0.05 compared with the SC-SAL group (+) and the SC-OVA group (*) (one-way ANOVA and post-hoc Holm-Sidak test). Values are the means ± S.E.M. and are representative of 1 experiment (n=5 per group).

Lung parenchyma of the control mice (SC-SAL) were normal (Figure 2A). Histological lung evaluations of the SC-OVA mice revealed a marked peribronchiolar eosinophil accumulation (Figure 2B) compared with the SC-SAL mice. The FO intake did not alter the lung parenchyma in the FO-SAL mice (Figure 2C) but markedly inhibited the tissue eosinophil infiltration in the FO-OVA mice (Figure 2D). Quantitative morphometric analyses of lung sections demonstrated that FO markedly inhibited tissue eosinophil infiltration (*P*<0.0001) (Figure 2E).

### Effect of FO administration on lung remodeling and mucus deposition

**Figure 3:**
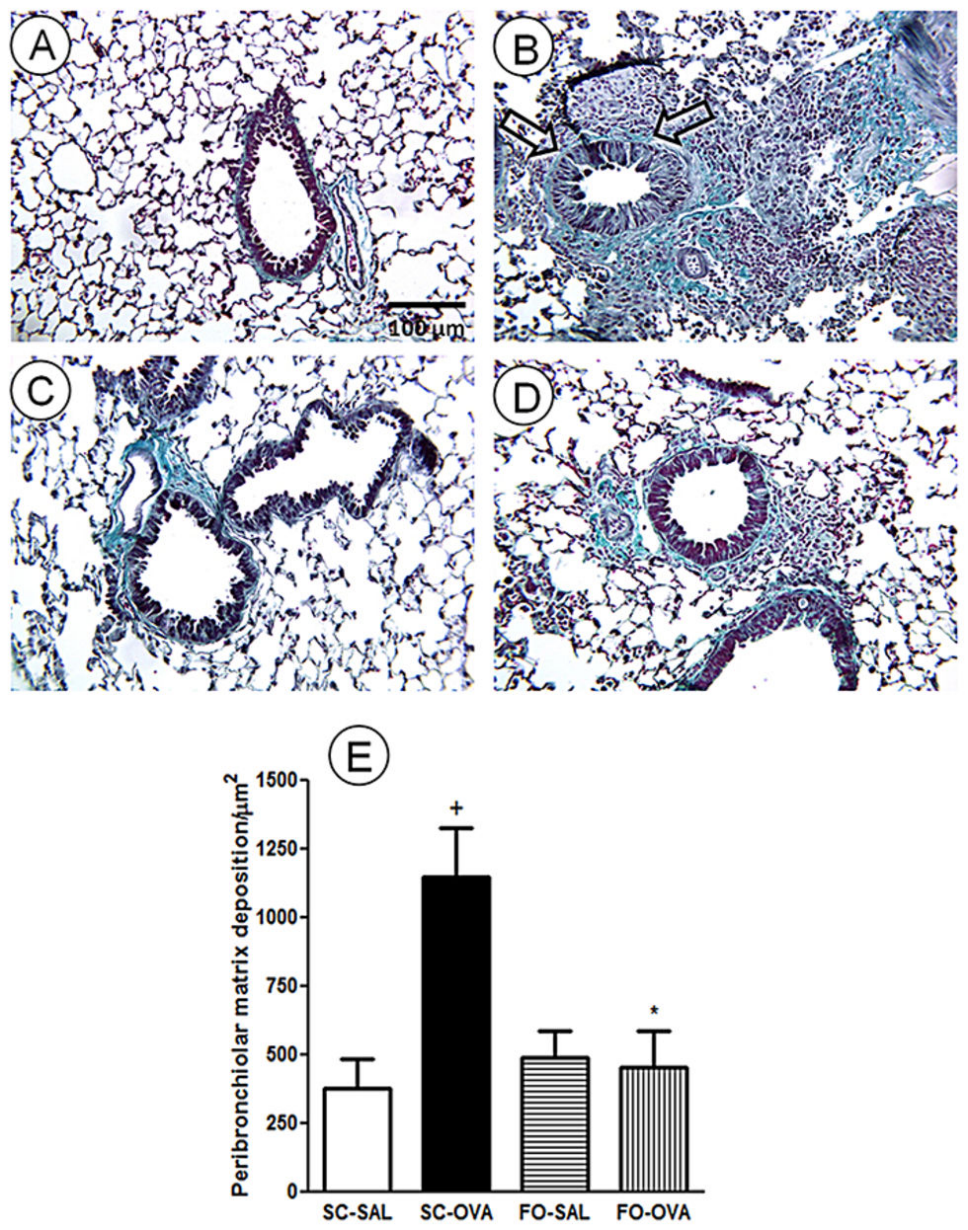
Effect of FO intake on allergen-evoked subepithelial fibrosis in A/J mouse lung tissue. Photomicrographs were taken of representative airways from (A) sensitized, saline-challenged (SC-SAL); (B) sensitized, ovalbumin-challenged (SC-OVA); (C) sensitized, saline-challenged with fish oil (FO-SAL) and (D) sensitized, ovalbumin-challenged with fish oil (FO-OVA). (E) Quantitative assessment of lung tissue fibrosis. The analyses were made 24 hours after the last challenge. The slides were stained with Gomori trichrome stain. Arrows indicate representative matrix deposition. In the signaled cases, *P*<0.05 compared with the SC-SAL group (+) and the SC-OVA group (*) (one-way ANOVA and post-hoc Holm-Sidak test). Values are the means ± S.E.M. and are representative of 1 experiment (n=5 per group).

As observed in Figure 3, lung sections stained with Gomori trichrome demonstrated that SC-SAL mice had normal lung parenchyma (Figure 3A) and that the SC-OVA mice had increased peribronchiolar matrix deposition (Figure 3B) compared with the SC-SAL mice (+ 205%, *P*=0.0051). No alterations were noted in the non-challenged mice that were given FO (FO-SAL) (Figure 3C). However, FO intake reduced responses in the FO-OVA mice (*P*=0.0099) (Figure 3D). Quantitative analyses demonstrated that FO intake prevented extracellular matrix deposition in the FO-OVA mice (Figure 3E).

**Figure 4:**
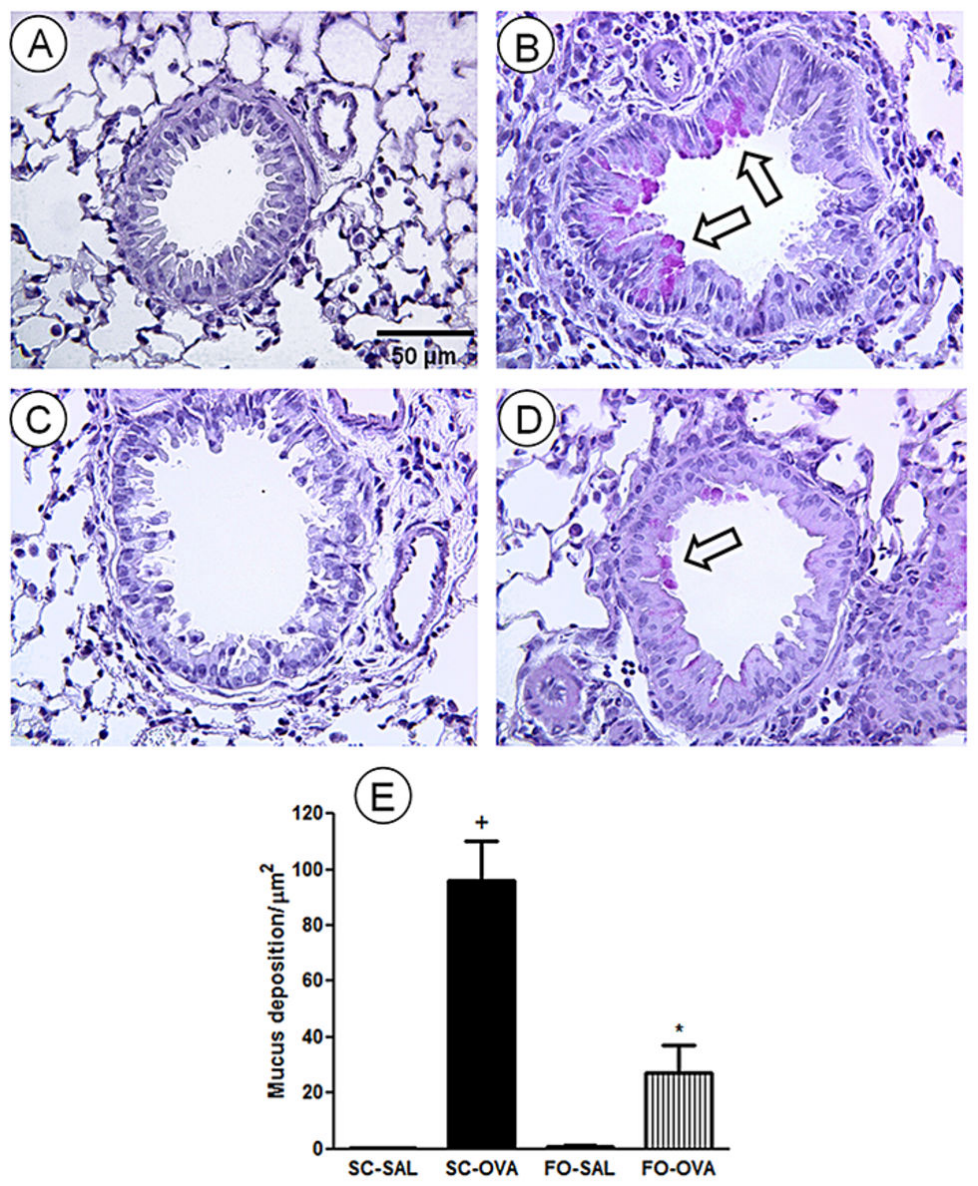
Effect of FO intake on allergen-evoked mucus production in A/J mouse lungs. Photomicrographs were taken of representative airways from (A) sensitized, saline-challenged (SC-SAL); (B) sensitized, ovalbumin-challenged (SC-OVA); (C) sensitized, saline-challenged with fish oil (FO-SAL) and (D) sensitized, ovalbumin-challenged with fish oil (FO-OVA). (E) Quantitative assessment of mucus production was performed by morphometric analyses. The analyses were performed 24 hours after the last challenge. Slides were stained with periodic acid-Schiff. Arrows indicate representative mucus deposition. In the signaled cases, *P*<0.05 compared with the SC-SAL group (+) and the SC-OVA group (*) (one-way ANOVA and post-hoc Holm-Sidak test). Values are the means ± S.E.M and are representative of 1 experiment (n=5 per group).

To evaluate mucus production, lung histology sections were stained with periodic acid-Schiff. SC-SAL and FO-SAL mice displayed no mucus production (Figure 4A and 4C, respectively), whereas SC-OVA mice had increased mucus secretion (*P*<0.0001) within the airway epithelia (Figure 4B). This phenomenon was prevented in the FO-OVA mice (-72%, *P*<0.0001) (Figure 4D). Quantitative analyses are shown in Figure 4E.

### Effect of FO administration on antigen-induced hyperreactivity (AHR)

**Figure 5:**
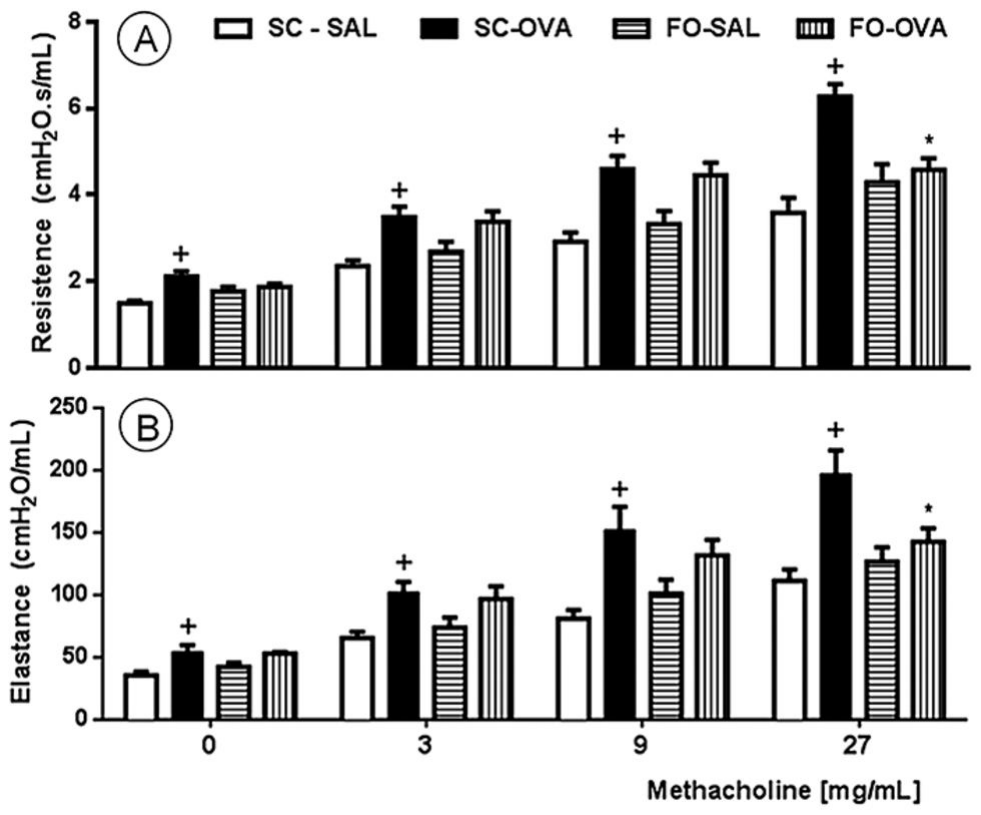
Effect of FO intake on allergen-induced changes in lung resistance (A) and elastance (B). Airway hyperreactivity was measured as changes that were induced by increasing methacholine concentrations 24 hours after the last antigen challenge. Sensitized, saline-challenged (SC-SAL); sensitized, ovalbumin-challenged (SC-OVA); sensitized, saline-challenged given fish oil (FO-SAL) and sensitized, ovalbumin-challenged with fish oil (FO-OVA). In the signaled cases, *P*<0.05 compared with the SC-SAL group (+) and the SC-OVA group (*) (one-way ANOVA and post-hoc Holm-Sidak test). Values are the means ± S.E.M. and are representative of 2 independent experiments (n=6 per group per experiment).

Antigen challenge of sensitized mice caused AHR, as demonstrated by increased lung resistance and elastance after methacholine (3-27 mg/mL) stimulation (Figure 5A and 5B, respectively) compared with control mice (SC-SAL). FO intake reduced AHR in the FO-OVA mice (Figure 5A and 5B).

### Effect of FO administration on serum specific anti-OVA IgE and IgG1

**Figure 6:**
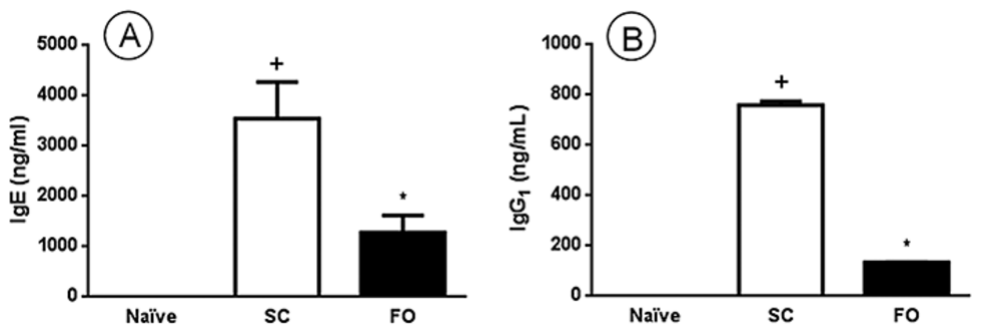
Effect of FO intake on allergen-induced serum IgE and IgG1 in A/J mice. Sensitized (SC) and sensitized, with fish oil (FO). In the signaled cases, *P*<0.05 compared with the Naive group (+) and the SC group (*) (one-way ANOVA and post-hoc Holm-Sidak test). Values are the means ± S.E.M. and are representative of 1 experiment (n=7 per group).

Naive animals showed no anti-OVA IgE and IgG1 in serum, meanwhile OVA-sensitized mice fed a SC-diet (SC group) demonstrated increased levels of both IgE and IgG1 (*P*=0.0001 and *P*<0.0001, respectively). Moreover, OVA sensitized mice fed a FO-diet (FO group) presented a reduction of 64% in serum IgE (*P*=0.0078) and of 83% in serum IgG1 when compared to SC group (*P*<0.0001). These results can be seen in Figure 6A and 6B.

### Effect of FO administration on lung cytokines

**Figure 7:**
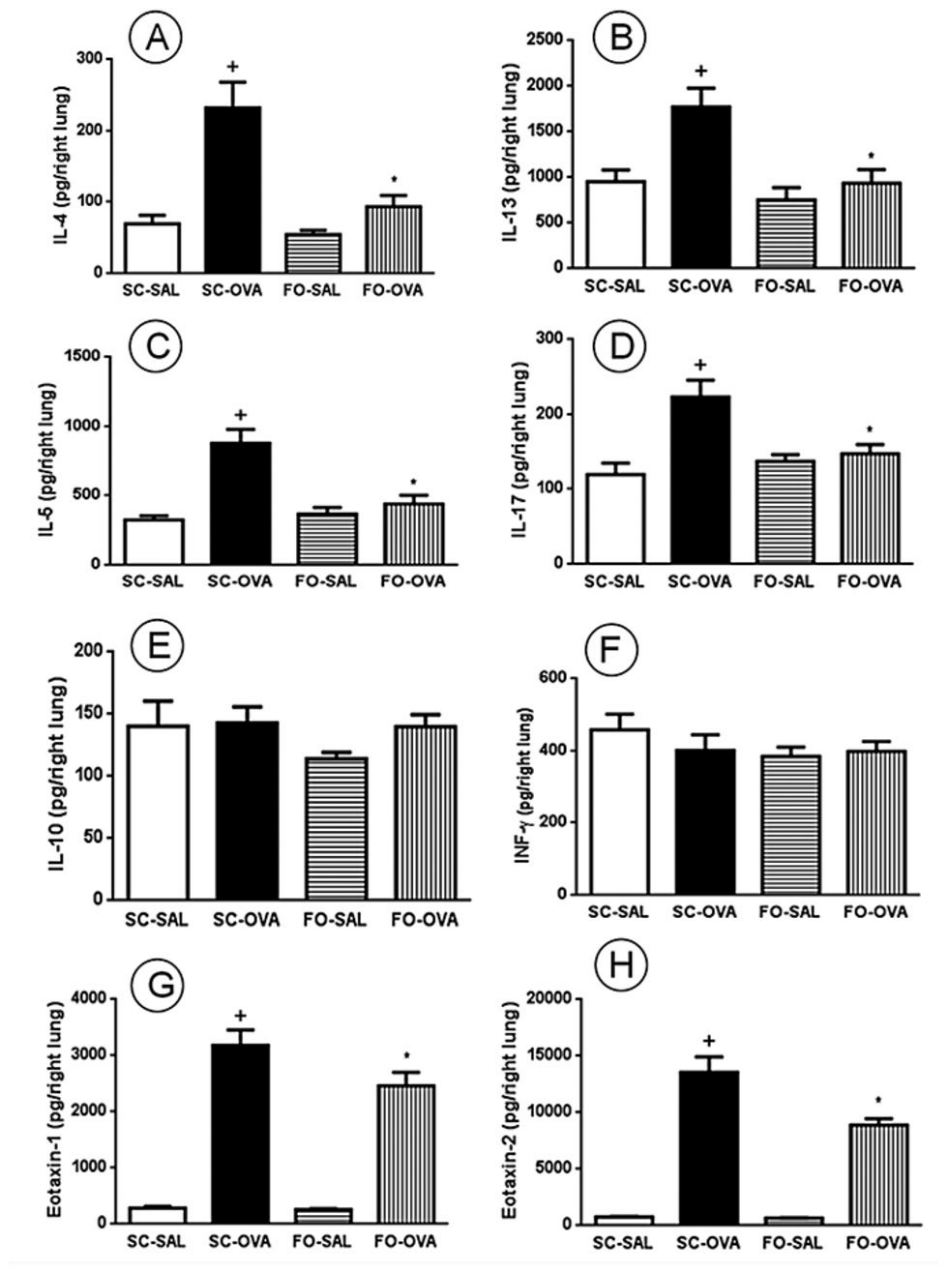
Effect of FO intake on allergen-induced IL-4 (A), IL-13 (B), IL-5 (C), IL-17 (D), IL-10 (E), INFγ (F), eotaxin-1 (G) -2 production (H), in the lung tissue of A/J mice, 24 hours after the last challenge. Sensitized, saline-challenged (SC-SAL); sensitized, ovalbumin-challenged (SC-OVA); sensitized, saline-challenged given fish oil (FO-SAL) and sensitized, ovalbumin-challenged with fish oil (FO-OVA). In the signaled cases, *P*<0.05 compared with the SC-SAL group (+) and the SC-OVA group (*) (one-way ANOVA and post-hoc Holm-Sidak test). Values are the means ± S.E.M. and are representative of 1 experiment (n=7 per group).

The lungs from OVA-challenged mice presented higher levels of IL-4 (Figure 7A), IL-5 (Figure 7C), IL-13 (Figure 7B), IL-17 (Figure 7D), eotaxin-1 (Figure 7G) and eotaxin-2 (Figure 7H) as compared to SAL-challenged animals. As shown in Figure 7, FO intake markedly reduced the levels of these pro-inflammatory and pro-fibrotic cytokines in the lung of OVA-challenge mice: IL-4 (-60%, *P*=0.0004), IL-5 (-50%, *P*=0.0002), IL-13 (-47%, *P*=0.0042), IL17 (-34%, *P*=0.0072), eotaxin-1 (-23%, *P*=0.0212) and eotaxin-2 (-35%, *P*=0.0004). No increase of IL-10 and INFγ levels were detected in the lungs of OVA-challenged mice compared to control group, and the treatment with FO did not significantly altered basal levels of both cytokines (Figure 7E and 7F).

### Effect of FO administration on NFκB, GATA-3 and PPARγ expression

**Figure 8:**
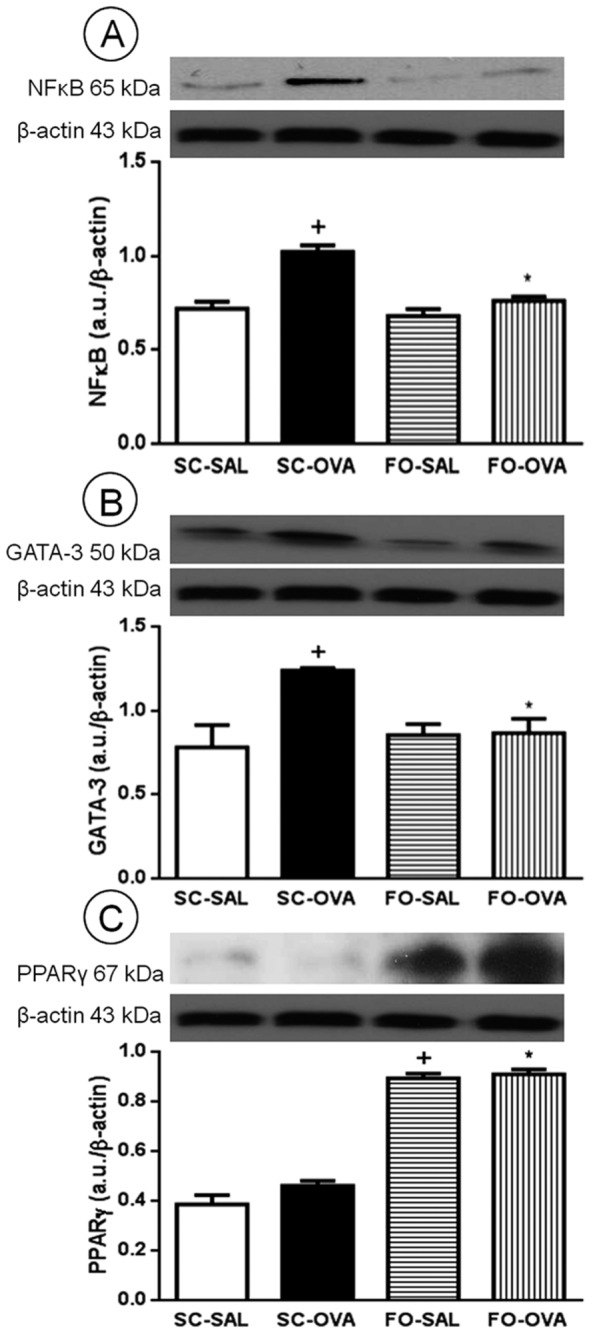
Effect of FO intake on nuclear factor kappa B (NFκB) (A), GATA-3 (B) and peroxisome proliferator-activated receptor gamma (PPARγ) (C) expression in A/J mouse pulmonary tissue. Sensitized, saline-challenged (SC-SAL); sensitized, ovalbumin-challenged (SC-OVA); sensitized, saline-challenged with fish oil (FO-SAL) and sensitized, ovalbumin-challenged with fish oil (FO-OVA). The results were standardized to β-actin expression and were expressed in arbitrary units (a.u.). The analyses were performed 24 hours after the last challenge. In the signaled cases, *P*<0.05 compared with the SC-SAL group (+) and the SC-OVA group (*) (one-way ANOVA and post-hoc Holm-Sidak test). Values are the means ± S.E.M. and are representative of 1 experiment (n=5 per group).

As illustrated in Figure 8A and 8B, the SC-OVA mice exhibited elevated NFκB and GATA-3 expression in whole-lung extracts compared with the SC-SAL mice (+42%, *P*=0.0002 and +59%, *P*=0.0162, respectively). FO-OVA mice had reduced NFκB (*P*=0.0006) (Figure 8A) and GATA-3(*P*=0.0410) expression (Figure 8B). FO-SAL and FO-OVA mice had increased PPARγ expression in lung extracts compared with the SC-SAL (+132%, *P*<0.0001) and SC-OVA (+98%, *P*<0.0001) mice (Figure 8C).

## Discussion

In this study, we demonstrated that FO intake before and during sensitization and challenge ameliorated the inflammatory response by reducing eosinophil infiltration in the BAL fluid and lung tissue, remodeling and mucus deposition, inflammatory and pro-fibrotic cytokine production, which contributed to diminished airway hyperreactivity. In addition, FO down-regulated serum anti-OVA IgE and IgG1 production and the expression of inflammatory transcription factors GATA-3 and NFκB.

In accordance with a previous study, ovalbumin-challenged mice showed an increase in the bronchoalveolar accumulation of leukocytes as compared to controls, a response which was accounted for by elevated number of mononuclear cells, neutrophils and eosinophils [20]. Moreover, pulmonary tissue eosinophil infiltration was increased in mice, confirming previous data from the literature [21,22]. Eosinophils are major disease effectors that contribute to the release of a variety of inflammatory mediators such as cytokines, chemokines, lipid mediators and cationic proteins [23,24]. Eosinophil infiltration can be controlled by IL-5, a Th2-type cytokine [25], which is present in lung tissue from OVA-challenged mice and causes induction of eosinophils maturation in bone marrow and eosinophil traffic to the site of inflammation [24,26]. It can also be controlled by eotaxin, which is comprised of eotaxin-1 (CCL11) and eotaxin-2 (CCL24) in mice [27]. These belong to a family called CC chemokines, which are produced by epithelial cells, alveolar macrophages and dendritic cells and bind to the eotaxin receptor (CCR3) in eosinophils [25]. Eotaxin-1 and 2 are elevated in lung tissue of allergen-challenged mice, are responsible for eosinophil influx and contribute to eosinophilia [25,26]. Increased levels of such mediators, as in this study, could explain the increased eosinophils in both the BAL fluid and the lung tissue. In our study, FO consumption decreased eosinophil infiltration, which is in contrast to previous observations regarding lung inflammation in mice that received FO by gavage for 14 days [15]. This response can be associated, at least in part, with reduction of IL-5 and eotaxin-1 and -2 generation.

Asthma is also characterized by structural changes in the airways, which includes subepithelial fibrosis, epithelial damage and smooth muscle hypertrophy [17]. In histological lung sections, we noted increased extracellular matrix deposition in the SC-OVA mice [17,21]. Subepithelial/peribronchial fibrosis is characterized by extracellular matrix protein deposits beneath the basal lamina, which is controlled by the cytokines such as IL-4 and IL-13 in asthma [28]. IL-4 and IL-13 orchestrate asthma-associated inflammation and are Th2 cytokines that are produced not only by lymphocytes but also by mast cells, eosinophils and macrophages. Both cytokines are induced in ovalbumin-challenged mice and are responsible for several structural and functional disease alterations [23,28]. Therefore, IL-4 and IL-13 reductions may be a mechanism for the decreased extracellular matrix deposition in the FO-OVA group.

Goblet cell hyperplasia and mucus hypersecretion are also important features of asthma. In our study we found elevated bronchiolar mucus deposition, confirming a previous study [21]. Airway goblet cells secrete mucin into the airway lumen in response to a variety of stimuli such as leukotrienes, IL-4 and IL-13. FO diminishes mucus deposition [15]. Thus, our findings of reduced IL-4 and IL-13 in the FO-OVA group could explain this reduced mucus secretion.

As expected, the ovalbumin-challenged mice (SC-OVA group) had elevated airway hyperreactivity, which is a major feature of asthma. AHR is an immoderate airway response to several allergens, although the causes underlying this condition are not well understood [29]. Some authors indicate that eosinophil infiltration has an important role in the development of AHR [30], while others demonstrate a close association between AHR and T helper lymphocytes [29]. Moreover, hyperreactivity is caused by a direct effect of IL-13 on airway smooth muscle [31]. In contrast to the study from Wood and colleagues, we report that FO intake improved resistance and elastance [15]. Because some possible AHR causes (i.e., leukocyte infiltration and alterations in IL-13-stimulated airway smooth muscle) are diminished in the FO-OVA mice, we expected reduced airway hyperreactivity in the mice that had consumed FO.

More recently, a distinct lineage of T helper cells has been described (Th17 cells) which produce a variety of cytokines, including IL-17 [32], which were shown to induce tissue remodeling and AHR [33]. Thus, diminished levels of IL-17 in lungs of FO-OVA group could explain the reduction of tissue remodeling and AHR.

The production of IL-4 and IL-13 by Th2 cells is known to control the further production of IgE antibody [34,35], which is known to bind to high affinity receptor (FceRI) mainly presented on mast cells [36]. These cells activation leads to the release of Th2-type cytokines such as IL-5 and IL13, which have the ability to induce eosinophil accumulation [35,37] by means of regulation of adhesion molecules expression and cell locomotory activity [36]. Besides that, IgG1 is also induced by Th2 cytokines like IL-4 and is cytophillic to mast cells [38,39]. In this study we showed that OVA allergic mice exhibited high levels of specific serum anti-OVA IgE and IgG1, a response suppressed under conditions of FO administration. The augmentation of serum levels of IgE and IgG1 under conditions of allergen sensitization has been shown previously [40–42]. Our data are in line with previous report showing that IgE production was reduced by FO in primary human B cells *in vitro* [10] and that both IgE and IgG1 were diminished after FO administration to OVA-sensitized mice [43]. The reduction in OVA-specific antibodies shows that FO affects the sensitization phase and that this could be a possible explanation for the reduced inflammatory response noted in FO-OVA group.

Mice that were genetically modified to produce greater amounts of endogenous n-3 PUFA displayed less leukocyte infiltration and mucus production in the lungs, lower total leukocyte and eosinophil number in the BAL fluid and reduced airway resistance [44]. In a previous study, using maternal protein restriction diet as a model, we observed that offspring that received FO in their diet (at the same dose of this study) during postnatal life had improved metabolic and morphological parameters in adulthood [45]. The FO dose that was given in this study was higher than that of other one using OVA-challenged mice, which demonstrated that FO might be incorporated into mouse lung tissue in a dose- and time-dependent manner [7]. FO includes 12.9% EPA and 12% DHA, totaling a content of almost 25% of these n-3 PUFA, normally seen in FO [11]. In addition, FO containing 18% EPA and 12% DHA was also given to animals and showed suppressive effect on allergen-induced oxidative stress [15], indicating that both percentages of EPA and DHA contribute to the beneficial effect of FO [46]. Thus, we can speculate that differences in the dose regimen and administration via may contribute to explain the discrepancy noted in our findings as compared to others.

There are few studies correlating EPA or DHA alone in preventing the development of allergic airway disease. The amelioration of bronchial hyperreactivity and cellular infiltration was noted under conditions of DHA aerosolization during challenge period. Another study using a DHA-derivate demonstrated beneficial effects in inflammatory response. In fact, a study with rhinovuris-infected cultured airway epithelial cells showed that only DHA had a potential role to suppress airway inflammation, while EPA presented no effects. On the other hand, in LPS-induced human asthmatic alveolar macrophage cells, the anti-inflammatory effects of EPA were much greater than effects of DHA. These data demonstrate that DHA seems to have a more expressive effect when compared to EPA despite lacking of consensus.

EPA and DHA can modulate inflammatory processes [47]. Eicosanoid lipid mediators are produced from cell membrane arachidonic acid degradation and have an important role in asthma. The 2-series prostaglandins (PG_2_) are produced by the enzyme cycloxygenase-2 (COX-2), and the 4-series leukotrienes (LT_4_) are produced by 5-lipoxygenase (5-LOX) [47]. PGD_2_ is secreted by mast cells and is important for the acute asthmatic airway response and inflammatory cell recruitment [48]. PGE_2_ has both inflammatory and anti-inflammatory effects [47]. LTB_4_ is implicated in chemoatraction and neutrophil activation [49]. Cysteinyl-leukotrienes (i.e., LTC_4_, LTD_4_ and LTE_4_) cause mucus hypersecretion and eosinophil airway infiltration [15]. N-3 PUFA intake alters cellular membrane fatty acid content [50], leading to decreased PG_2_ and LT_4_ production and enhanced PG_3_ and LT_5_ production, which has less potent inflammatory activity [11,14]. LTB_5_ has less chemotactic activity and fewer aggregating properties than LTB_4_ [14]. In addition, PGD_3_ inhibits the action of PGD_2_ [51]. In our study, we hypothesized that FO intake altered the cell membrane lipid profile and consequently the proportional production of PG_2_-LT_4_/PG_3_-LT_5_, thus decreasing the response to allergen.

In this study we showed that treatment with FO did not cause any alteration in the levels of IL-10 and INFγ in the lung tissue of OVA-challenged mice, indicating that the suppressive effect of FO on the allergic lung response in mice does not seem to be dependent on the production of the anti-inflammatory cytokines or to a shift towards a Th1 prolife.

EPA and DHA, in addition to their effects mentioned above, directly control the expression of transcription factors [11]. GATA-3 is responsible for the development and differentiation of CD4^+^ lymphocytes [52]. DHA diminished GATA-3 expression in a mouse model of experimental autoimmune encephalomyelitis [53], in accordance with our data. NFκB is expressed in inflammatory cells, and its activation causes the production of several inflammatory proteins including cytokines and COX-2. To be activated, NFκB must be disassociated from its inhibitory subunit IκB by phosphorylation [54]. FO consumption decreases IκB phosphorylation and diminishes NFκB activation and inflammatory protein production [55]. In our study, we found that elevated NFκB expression in OVA-challenged mice could explain the increase in inflammatory cytokine and eicosanoid expression in these animals. Moreover, FO appears to modulate NFκB expression in the FO-OVA group, attenuating inflammatory response.

PPARγ can also be modulated by FO [11]. Recent studies demonstrated that PPARγ agonists reduce AHR, eosinophilia and Th2 cytokine and chemokine levels [56,57]. PPARγ binds to NFκB, thus blocking its nuclear translocation and inhibiting its pro-inflammatory properties [58]. It could be postulated that FO intake diminished inflammatory mediator production by reduced NFκB activation and increased PPARγ activity in this experiment.

In conclusion, our results show that prophylactic FO intake reduced airway hyperreactivity and impaired eosinophil inflammation, mucus production, peribronchiolar fibrosis and cytokine production in sensitized antigen-challenged mice using a mechanism that is associated with down-regulation of NFκB and GATA-3 and the up-regulation of PPARγ expression. OVA-specific serum IgE and IgG1 were also sensitive to FO. Altogether, our findings show that FO has beneficial effect to prevent systemic sensitization, and indicate that FO can be considered as a potent new prophylactic adjuvant for the asthma prevention.
